# Pigmentation pattern and developmental constraints: flight muscle attachment sites delimit the thoracic trident of ***Drosophila melanogaster***

**DOI:** 10.1038/s41598-018-23741-4

**Published:** 2018-03-28

**Authors:** Jean-Michel Gibert, Emmanuèle Mouchel-Vielh, Frédérique Peronnet

**Affiliations:** Sorbonne Université, CNRS, Biologie du Développement Paris Seine-Institut de Biologie Paris Seine (LBD-IBPS), UMR7622, Team “Epigenetic control of developmental homeostasis and plasticity”, 75005 Paris, France

## Abstract

In their seminal paper published in 1979, Gould and Lewontin argued that some traits arise as by-products of the development of other structures and not for direct utility in themselves. We show here that this applies to the trident, a pigmentation pattern observed on the thorax of *Drosophila melanogaster*. Using reporter constructs, we show that the expression domain of several genes encoding pigmentation enzymes follows the trident shape. This domain is complementary to the expression pattern of *stripe (sr)*, which encodes an essential transcription factor specifying flight muscle attachment sites. We demonstrate that *sr* limits the expression of these pigmentation enzyme genes to the trident by repressing them in its own expression domain, *i.e*. at the flight muscle attachment sites. We give evidence that repression of not only *yellow* but also other pigmentation genes, notably *tan*, is involved in the trident shape. The flight muscle attachment sites and *sr* expression patterns are remarkably conserved in dipterans reflecting the essential role of *sr*. Our data suggest that the trident is a by-product of flight muscle attachment site patterning that arose when *sr* was co-opted for the regulation of pigmentation enzyme coding genes.

## Introduction

Body pigmentation is at the interface between the organisms and their environment and fulfils many ecologically relevant functions. Indeed, the adaptive role of pigmentation seems often so obvious – *i.e*. in crypsis, mimicry, aposematism, mate recognition, UV protection or thermoregulation - that it is generally assumed that the observed patterns have been selected. Direct selection on pigmentation has been demonstrated in many cases, such as the famous industrial melanism of the peppered moth, *Biston betularia*, whose molecular basis has recently been identified^1,2^. Pigmentation patterns can also be the object of a trade-off between opposite selection forces. For example, in guppy *Poecilia reticulata* males, pigmentation patterns result from a balance between selection for crypsis, an anti-predator strategy, and selection for conspicuousness to attract females^3^. Further evidence for selection is the convergent loss of pigmentation when selection is relaxed in organisms living in the absence of light, such as cave animals^4,5^. However, since Gould and Lewontin’s seminal paper in 1979^6^, it is widely acknowledged that adaptationist explanations should sometime be used with caution and developmental constraints also taken into account. Gould and Lewontin used the example of the spandrels decorated with splendid mosaics between the arches supporting the dome of the basilica of Saint-Mark in Venice. Although the mosaics fit remarkably well on the spandrels, the spandrels were not designed for them but result from architectural constraints imposed by the structure supporting the dome. Thus, Gould and Lewontin argued that some biological traits arose as by-products of developmental constraints on a crucial trait and were not selected for their direct utility^6,7^. Hence, developmental constraints favour particular patterns or morphologies whereas they forbid others. A few studies on pigmentation have addressed this question by exploring morphospaces with artificial selection experiments^8,9^. The size of the eyespots on butterfly *Bicyclus anynana* wings responds to selection but their colour is more constrained as only coordinated changes of pigments are possible^8^. Consequently, only some area of the morphospace can be occupied. Furthermore, the eyespot pattern through the genus *Bicyclus* follows a similar path of diversification, suggesting that evolution is partly constrained and that selection (or drift) can operate only in particular directions^8^.

*Drosophila* pigmentation is an interesting model to study the impact of developmental constraints^10^. This highly evolvable trait has been the focus of many studies analysing the genetic bases of morphological variation within and between species^11,12^. Body or wing pigmentation relies on the coordinated action of *trans*-regulatory factors on genes encoding pigmentation enzymes^13–20^. Most of these factors, for example Engrailed or Abdominal-B, are spatially restricted components of a deeply conserved regulatory landscape involved in development of many essential traits. Hence, pigmentation patterns can be interpreted as targets of indirect selection due to their association with another trait^21^.

The trident is a melanic pattern observed on the dorsal thorax of *Drosophila melanogaster*^22^. In natural populations, the intensity of the trident shows clinal genetic variation, with darker tridents observed at higher latitudes in Europe, India and Australia and higher altitudes in India and Africa^23–26^. The variable intensity of the trident is therefore thought to be an adaptation to temperature and/or UV^24,25^. Differences in the intensity of the trident were shown to be linked to genetic variation in the pigmentation gene *ebony (e)*, which is less expressed in flies with a darker trident^26,27^. Besides, the trident is more clearly visible in an *e* mutant background^28^. Furthermore, the intensity of the trident is sensitive to developmental temperature^24^. This trait is therefore an example of phenotypic plasticity, “*the ability of a given genotype to produce different phenotypes in response to distinct environmental conditions*”^29^. Interestingly, in *Drosophila busckii*, a pattern similar to the trident is very clearly delineated on the thorax, being dark brown on a yellow background^22,30^. Thus, the intensity of the trident is variable, plastic and highly evolvable. By contrast, the developmental bases of its shape have attracted little attention. Regulatory genes, whose mutation modifies it, are the most promising candidates to address the developmental constraints exerted. Mutation in the *stripe* gene (*sr*^*1*^ allele) affects the shape of the trident^31^. This gene encodes an Egr-like zinc-finger transcription factor specifying epidermal tendon cells, to which muscles attach^32–35^. Therefore, *sr* expression labels the flight muscle attachment sites on the pupal thorax^32,35^. The expression pattern of *sr* on the pupal thorax^36^ seems complementary to the trident, which suggests that *sr* might delimit the trident by repressing melanin production on the thorax. Using reporter constructs made with the regulatory sequences of pigmentation enzyme coding genes, we investigate here the role of *sr* in the establishment of the trident’s pattern and its possible relationship with the positioning of the flight muscles. Using mutants, we show that *sr* represses the expression of several pigmentation enzyme-coding genes in the thorax epidermis, thus shaping the trident. The flight muscle attachment sites and *sr* expression pattern are remarkably conserved in dipterans, reflecting the essential role of *sr*^36,37^. Our data suggest that the shape of the trident is a by-product of flight muscle attachment site patterning that arose when *sr* was co-opted for the regulation of pigmentation enzyme-coding genes.

## Results and Discussion

### *stripe* represses melanin production on the thorax

**Figure 1:**
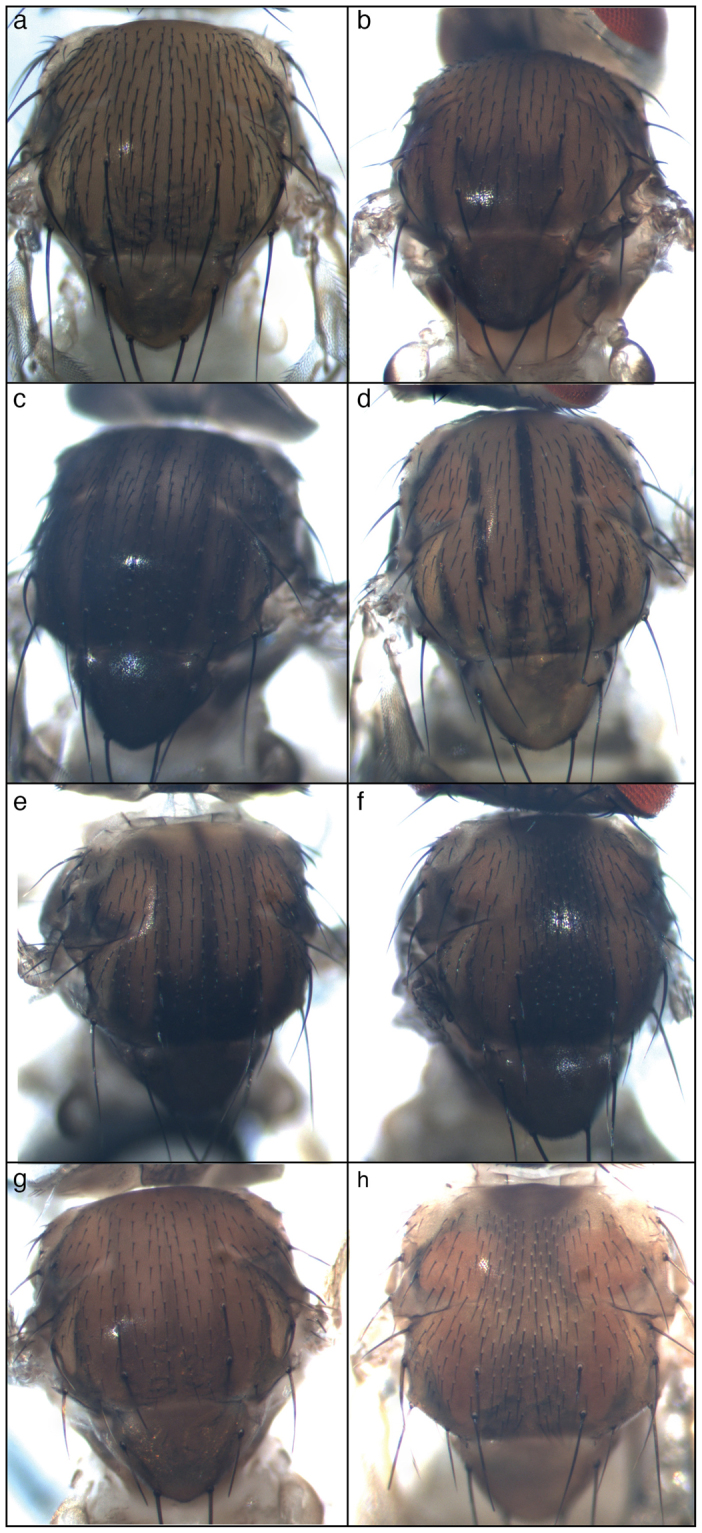
The trident pattern in *Drosophila*. (**a**) Absence of trident in *w*^*1118*^. (**b**) Trident clearly visible in *41Jd*. (**c**) Dark trident of an *ebony e*^*1*^ mutant. (**d**) Distinctive trident in *Drosophila busckii*. (**e**) Trident of an *ebony e*^*s*^ mutant. (**f**) Dark longitunal stripe in an *e*^*s*^ *sr*^*1*^ double mutant. (**g**) Trident still visible in an *y*^*1*^, *e*^*s*^ mutant background. (**g**) Longitudinal dark band in a *y*^*1*^*; e*^*s*^ *sr*^*1*^ mutant.

The pattern of the trident varies among *Drosophila melanogaster* lines, being absent in some of them (*w*^*1118*^) whereas clearly visible in others (*41Jd)* (Fig. 1a,b). In *ebony* mutant (*e*^*1*^), the trident is very visible (Fig. 1c) as previously reported^28^. The trident is not limited to *Drosophila melanogaster* as it is reminiscent of the pigmentation pattern observed in another *Drosophila* species, *Drosophila busckii* (Fig. 1d).

Apparent complementarity between *sr* expression pattern and the trident in the pupal thorax^36^ suggests that *sr* might be involved in trident patterning. To address this question, we took advantage of the *sr*^*1*^ allele of *sr*. This is a very old allele, as it is reported to have been isolated by Calvin Bridges (1889–1938)^31^. In *sr*^*1*^ flies, the trident is replaced by a broad longitudinal stripe, hence the name of the gene^31^. It is only thanks to the much more recent description of the *sr* expression pattern and to the characterization of the *sr*^*1*^ allele that this phenotype can be interpreted. It was shown that *sr*^*1*^ is a regulatory mutant, which loses the most dorsal domains of *sr* expression on the thorax^36^. To render the dark longitudinal stripe more visible, we combined *sr*^*1*^ with the *e*^*s*^ hypomorphic allele^31^. The dark dorsal longitudinal stripe induced by *sr*^*1*^ was perfectly visible in the *e*^*s*^ background (compare Fig. 1e,f). These data suggest that the dark longitudinal stripe is a modified trident, in which spaces between teeth are filled with melanin.

These spaces correspond to the dorsal domains of *sr* expression that are missing in *sr*^*1*^, which suggests that *sr* shapes the trident by repressing melanin production. This repression could occur through different mechanisms implicating the expression, the stability or/and the activity of one or several pigmentation enzymes. Production of cuticle pigments involves many enzyme-coding genes arranged into a pathway^38,39^. Interestingly, among them, *ple* (encoding the Tyrosine Hydroxylase), *Ddc* (encoding the Dopa decarboxylase), *yellow (y)*, and *e* were shown previously to be expressed in the trident^28,40,41^. Over-expression of *y* in the dorso-medial domain in an *e* mutant background is sufficient to generate a homogenous black pigmentation^28^. Thus, restriction of *y* expression to the trident is sufficient to explain its delimitation. However, in a *y*^1^, *e*^*s*^ double mutant, the trident is still visible (Fig. 1g) as previously reported^28^ and in a *y*^*1*^*; sr*^*1*^, *e*^*s*^ triple mutant, the longitudinal pattern typical of *sr*^*1*^ is clearly visible (Fig. 1h). This implies that other pigmentation enzymes than *y* and *e* are involved in the patterning of the trident downstream of *sr*.

### Expression of *stripe* and *tan* in the thorax are complementary

**Figure 2:**
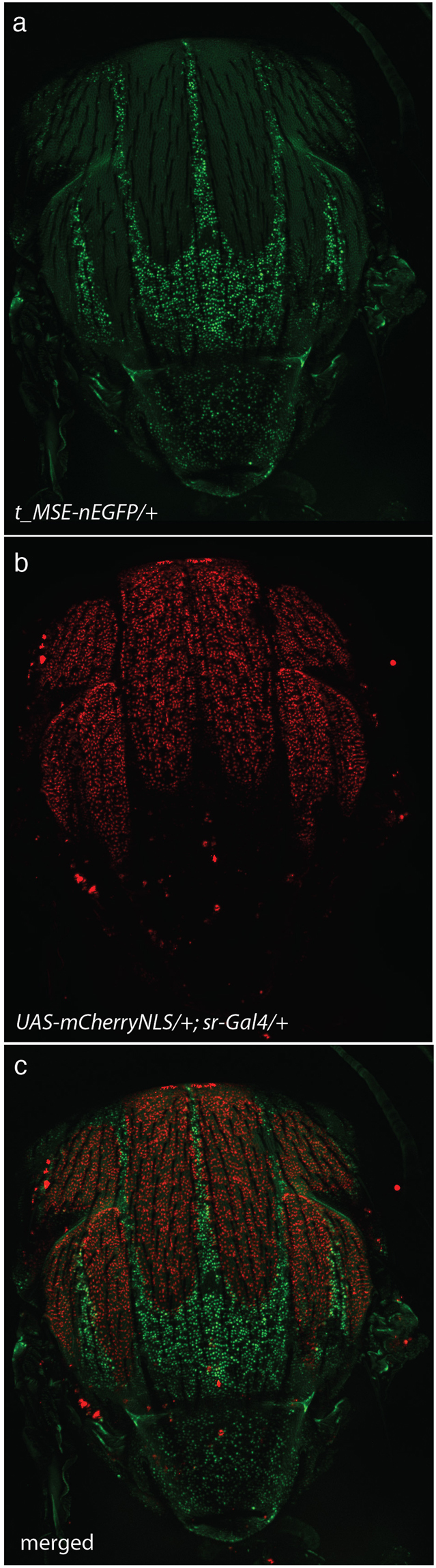
Complementary expression of *stripe* and *tan* in the thorax. Thorax of a *UAS-mCherry-NLS/t_MSE-nEGFP; sr-Gal4/*+ freshly eclosed fly. (**a**) nEGFP showing the activity of *t* regulatory sequences; (**b**) mCherry showing *sr* expression; (**c**) Merge.

Most pigmentation enzyme genes were previously shown to be expressed in the trident^28,40,41^. However, expression of *tan* (*t)*, which encodes an enzyme involved in melanin synthesis, was never analysed in the thorax. Using a transgene, in which the expression of nuclear enhanced green fluorescent protein (nEGFP) was driven by the *t* abdominal enhancer *t_MSE*^42,43^, we observed nEGFP expression in the trident (Fig. 2a), showing than *t_MSE* was also activated in this motif. To reveal *sr* expression, we used the enhancer trap line *sr*^*md710*^ *(sr-Gal4)*^36^ and the *UAS-mCherry-NLS* transgene. *mCherry* expression was visible in the notum of pupae, where it precisely labelled the flight muscle attachment sites^36^ (Fig. 2b). By combining (*sr*^*md710*^, *UAS-mCherry-NLS*) with the *t_MSE-nEGFP* transgene, we observed a remarkable complementarity between the patterns of mCherry and nEGFP (Fig. 2c). All flight muscle attachment sites expressing mCherry corresponded to regions where nEGFP was absent, and a high nEGFP level was observed outside of the flight muscle attachment sites.

### *stripe* delimits the shape of the trident by repressing multiple pigmentation genes

**Figure 3:**
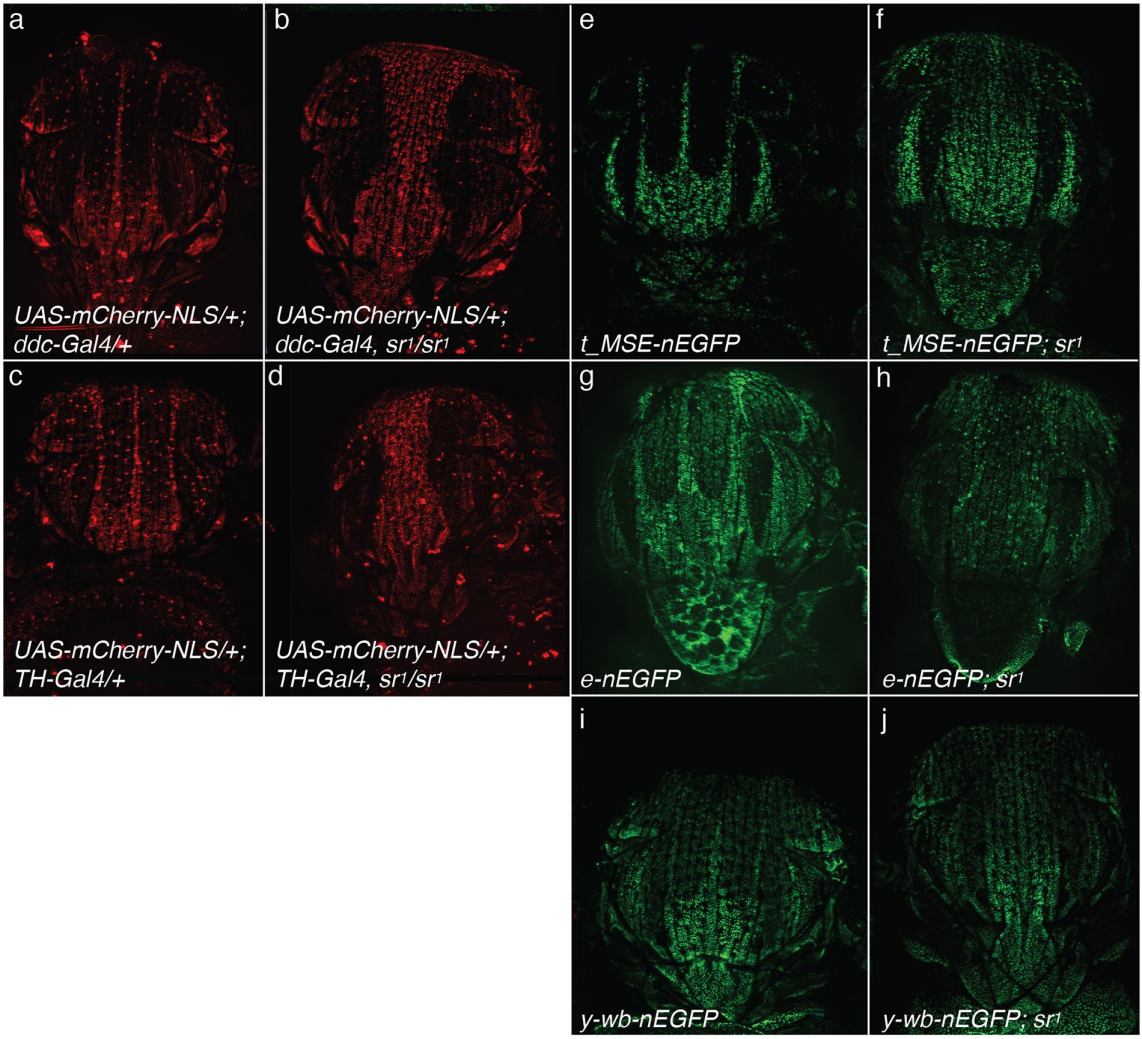
Effect of the *sr*^*1*^ mutation on the expression of pigmentation enzyme coding genes. (**a**) *Ddc* expression in thorax of pharates visualized using *Ddc-Gal4* and *UAS-mCherry-NLS* transgenes (*UAS-mCherry-NLS/*+*; Ddc-Gal4*). (**b**) *Ddc* expression in a *sr*^*1*^ background (*UAS-mCherry-NLS/*+; *Ddc-Gal4*, *sr*^*1*^*/sr*^*1*^). (**c**) *ple* expression visualized using *ple-Gal4* and *UAS-mCherry-NLS* transgenes (*UAS-mCherry-NLS/*+; *ple-Gal4/*+). (**d**) *ple* expression in a *sr*^*1*^ background (*UAS-mCherry-NLS/*+; *ple-Gal4*, *sr*^*1*^*/sr*^*1*^). (**e**) *t* expression followed using the *t_MSE-nEGFP* transgene. (**f**) *t* expression in a *sr*^*1*^ background (*t-MSE-nEGFP; sr*^*1*^). (**g**) *e* expression visualized using the *e-nEGFP* transgene. (**h**) *t* expression in a *sr*^*1*^ background (*e-nEGFP*, *sr*^*1*^*/sr*^*1*^). (**i**) *y* expression followed using the *y-wb-nEGFP* transgene. (**j**) *y* expression in a *sr*^*1*^ background (*y-wb-nEGFP; sr*^*1*^).

Complementarity between *sr* and *t* expression domains on the thorax suggests that *sr* might repress the expression of *t*. This could also be the case for all pigmentation enzyme genes. Then, to draw up a precise and complete analysis of the expression domains of pigmentation enzymes in the thorax, as compared to the expression domain of *sr*, we used *Ddc-Gal4*^44^ and *ple-Gal4*^45^ associated with the *UAS-mCherry-NLS* transgene as well as transgenes expressing *nEGFP* under the control of *t*, *e* or *y* regulatory sequences^13,41,42^. In the control background, all reporters were expressed in the thorax with patterns that resembled the trident (Fig. 3a,c,e,g,i) suggesting that the expression of pigmentation enzyme genes was constrained to this motif. By contrast, in the *sr*^*1*^ background, expression of the reporters was extended dorsally and the trident motif disappeared (Fig. 3b,d,f,h,j as compared to a, c, e, g, i, respectively). These data indicate that *sr* represses *Ddc*, *ple*, *t*, *e* and *y* in the thorax. Hence, the shape of the trident likely reflects the spatial regulation of pigmentation enzyme coding genes by Stripe.

## Conclusion

We show here that *sr* regulates several pigmentation enzyme-coding genes on the thorax, although we do not know whether this regulation is direct or not. The expression of *sr* is conserved in *Calliphora vicina*, a species that diverged from *Drosophila* lineage about 100 million years ago, suggesting that this gene is a member of a deeply conserved regulatory landscape^37^. *sr* plays an essential role in the establishment of flight muscle attachment sites, and conservation of its expression is mirrored by a remarkable conservation of the flight muscle apparatus in dipterans^46^. In contrast, a clear thoracic trident complementary to the flight muscle attachment sites is observed in only a few species of flies, notably in *Drosophila melanogaster*^22^. Our results suggest that the co-option of *sr* for the regulation of pigmentation enzyme coding genes has led to the generation of the trident, a pigmentation pattern complementary to the flight muscle attachment sites. Therefore, the shape of the trident primarily results from a developmental constraint imposed by the flight muscle pattern. It is typically a “spandrel” in the sense of Gould and Lewontin (1979), a by-product of the development of another structure. The same applies to the position of large bristles (macrochaetes) on the thorax. Indeed, macrochaetes are excluded from the flight muscle attachment sites, and it was shown that the development of macrochaetes and tendon cells on the thorax are mutually exclusive^36^.

The fact that the trident was originally an indirect target of selection does not exclude that it has later become a direct target of selection. Indeed, clinal variation in the intensity of the trident strongly suggests an adaptive role in thermoregulation^23,24,26^. However, natural selection has targeted variation in the intensity of the trident, rather than in its shape that is highly constrained by fixed muscle attachment sites. Furthermore, it is possible that the expression of pigmentation enzymes in the trident confers new properties to the thorax cuticle that are important for flight, such as flexibility or mechanical endurance.

## Methods

### Fly stocks

*w*^*1118*^ is an inbred line used as control. The line *41Jb* was established by Jean-Michel Gibert from a female caught in Marsais (France). The *Drosophila busckii* line was established from a female caught in Niort (France) and kindly provided by Dr Laure Teysset. The following stocks were obtained from the Bloomington Drosophila stock centre: *e*^*1*^ (BL-1658), *e*^*s*^ (BL-498), *Ddc-Gal4* (BL-7009), *ple-Gal4* aka *TH-Gal4* (BL-8848), *sr-Gal4* aka *sr*^*md710*^ (BL-26663) and *UAS-mCherry-NLS* (BL-38425). The lines *y-wing-body-nEGFP*, *e-nEGFP (*containing the regulatory regions *ABC* + *intron)* and *t_MSE-nEGFP* were kindly provided by Dr. Sean Carroll’s laboratory. Flies were grown on standard medium at 25 °C.

### Image acquisitions

Thoracic cuticles of flies immerged in 75% ethanol were imaged with a binocular equipped with Leica DC480 digital camera, using the Leica IM50 Image Manager software. Stacks of 4–10 images were generated for each thorax. They were merged using Photoshop. Identical settings were used for all acquisitions.

Fluorescent images were acquired with a Macro-Apotome (Zeiss) with a 63 × objective on freshly decapitated flies immerged in PBS on an agarose substrate. Stacks were composed of around 75–115 pictures. Maximum intensity projections were created. Brightness and contrast were slightly adjusted in Photoshop.

### Data availability

Data and materials used in this work are available on request.

## References

[1] Van’t Hof AE (2016). The industrial melanism mutation in British peppered moths is a transposable element. Nature.

[2] Nadeau NJ (2016). The gene cortex controls mimicry and crypsis in butterflies and moths. Nature.

[3] Endler JA (1980). Natural selection on color patterns in *Poecilia reticulata*. Evolution.

[4] Casane D, Rétaux S (2016). Evolutionary Genetics of the Cavefish *Astyanax mexicanus*. Adv. Genet..

[5] Bilandžija H, Cetković H, Jeffery WR (2012). Evolution of albinism in cave planthoppers by a convergent defect in the first step of melanin biosynthesis. Evol. Dev..

[6] Gould SJ, Lewontin RC (1979). The spandrels of San Marco and the Panglossian paradigm: a critique of the adaptationist programme. Proc. R. Soc. Lond. B Biol. Sci..

[7] Gould SJ (1997). The exaptive excellence of spandrels as a term and prototype. Proc. Natl. Acad. Sci. USA.

[8] Allen CE, Beldade P, Zwaan BJ, Brakefield PM (2008). Differences in the selection response of serially repeated color pattern characters: standing variation, development, and evolution. BMC Evol. Biol..

[9] Beldade P, Koops K, Brakefield PM (2002). Developmental constraints versus flexibility in morphological evolution. Nature.

[10] Gompel N, Carroll SB (2003). Genetic mechanisms and constraints governing the evolution of correlated traits in drosophilid flies. Nature.

[11] Wittkopp PJ, Carroll SB, Kopp A (2003). Evolution in black and white: genetic control of pigment patterns in *Drosophila*. Trends Genet.

[12] Massey JH, Wittkopp PJ (2016). The Genetic Basis of Pigmentation Differences Within and Between *Drosophila* Species. Curr. Top. Dev. Biol..

[13] Jeong S, Rokas A, Carroll SB (2006). Regulation of body pigmentation by the Abdominal-B Hox protein and its gain and loss in *Drosophila* evolution. Cell.

[14] Arnoult L (2013). Emergence and diversification of fly pigmentation through evolution of a gene regulatory module. Science.

[15] Gompel N, Prud’homme B, Wittkopp PJ, Kassner VA, Carroll SB (2005). Chance caught on the wing: *cis*-regulatory evolution and the origin of pigment patterns in *Drosophila*. Nature.

[16] Prud’homme B (2006). Repeated morphological evolution through *cis-*regulatory changes in a pleiotropic gene. Nature.

[17] Koshikawa S (2015). Gain of *cis*-regulatory activities underlies novel domains of *wingless* gene expression in. Drosophila. Proc. Natl. Acad. Sci. USA.

[18] Werner T, Koshikawa S, Williams TM, Carroll SB (2010). Generation of a novel wing colour pattern by the Wingless morphogen. Nature.

[19] Williams TM (2008). The regulation and evolution of a genetic switch controlling sexually dimorphic traits in *Drosophila*. Cell.

[20] Bray MJ, Werner T, Dyer KA (2014). Two genomic regions together cause dark abdominal pigmentation in *Drosophila tenebrosa*. Heredity.

[21] Pigliucci, null & Kaplan, null. The fall and rise of Dr Pangloss: adaptationism andthe Spandrels paper 20 years later. T*rends Ecol. Evol*. 1**5**, 66–70 (2000). 10.1016/s0169-5347(99)01762-0 10652558

[22] Werner, T. & Jaenike, J. *Drosophilids of the Midwest and Northeast*. (River Campus Libraries, University of Rochester).

[23] Munjal AK (1997). Thoracic trident pigmentation in *Drosophila melanogaster*: latitudinal and altitudinal clines in Indian populations. Genet Sel Evol.

[24] David JR, Capy P, Payant V, Tsakas S (1985). Thoracic trident pigmentation in *Drosophila melanogaster*: Differentiation of geographical populations. Genet Sel Evol.

[25] Bastide H, Yassin A, Johanning EJ, Pool JE (2014). Pigmentation in *Drosophila melanogaster* reaches its maximum in Ethiopia and correlates most strongly with ultra-violet radiation in sub-Saharan Africa. BMC Evol Biol.

[26] Telonis-Scott M, Hoffmann AA, Sgro CM (2011). The molecular genetics of clinal variation: a case study of *ebony* and thoracic trident pigmentation in *Drosophila melanogaster* from eastern Australia. Mol Ecol.

[27] Takahashi A, Takahashi K, Ueda R, Takano-Shimizu T (2007). Natural variation of *ebony* gene controlling thoracic pigmentation in *Drosophila melanogaster*. Genetics.

[28] Wittkopp PJ, True JR, Carroll SB (2002). Reciprocal functions of the *Drosophila* yellow and ebony proteins in the development and evolution of pigment patterns. Development.

[29] Pigliucci, M. *Phenotypic Plasticity, Beyond Nature and Nurture*. (2001).

[30] Shorrocks, B. *Drosophila*. (Ginn & company limited, 1972).

[31] Lindsley, D. L. & Zimm, G. G. *The genome of Drosophila melanogaster*. (Academic Press Limited, 1992).

[32] Fernandes JJ, Celniker SE, VijayRaghavan K (1996). Development of the indirect flight muscle attachment sites in *Drosophila*: role of the PS integrins and the *stripe* gene. Dev Biol.

[33] Frommer G, Vorbrüggen G, Pasca G, Jäckle H, Volk T (1996). Epidermal egr-like zinc finger protein of *Drosophila* participates in myotube guidance. EMBO J..

[34] Lee JC, VijayRaghavan K, Celniker SE, Tanouye MA (1995). Identification of a *Drosophila* muscle development gene with structural homology to mammalian early growth response transcription factors. Proc. Natl. Acad. Sci. USA.

[35] Ghazi A, Paul L, VijayRaghavan K (2003). Prepattern genes and signaling molecules regulate stripe expression to specify *Drosophila* flight muscle attachment sites. Mech. Dev..

[36] Usui K, Pistillo D, Simpson P (2004). Mutual exclusion of sensory bristles and tendons on the notum of dipteran flies. Curr Biol.

[37] Richardson J, Simpson P (2006). A conserved *trans*-regulatory landscape for *scute* expression on the notum of cyclorraphous Diptera. Dev. Genes Evol..

[38] Wright TR (1987). The genetics of biogenic amine metabolism, sclerotization, and melanization in *Drosophila melanogaster*. Adv. Genet..

[39] Gibert J-M, Mouchel-Vielh E, Peronnet F (2017). Modulation of *yellow* expression contributes to thermal plasticity of female abdominal pigmentation in *Drosophila melanogaster*. Sci. Rep..

[40] Gibert JM, Peronnet F, Schlotterer C (2007). Phenotypic Plasticity in *Drosophila* Pigmentation Caused by Temperature Sensitivity of a Chromatin Regulator Network. PLoS Genet.

[41] Rebeiz M, Pool JE, Kassner VA, Aquadro CF, Carroll SB (2009). Stepwise modification of a modular enhancer underlies adaptation in a *Drosophila* population. Science.

[42] Jeong S (2008). The evolution of gene regulation underlies a morphological difference between two *Drosophila* sister species. Cell.

[43] Gibert J-M, Mouchel-Vielh E, De Castro S, Peronnet F (2016). Phenotypic Plasticity through Transcriptional Regulation of the Evolutionary Hotspot Gene tan in *Drosophila melanogaster*. PLoS Genet..

[44] Li H, Chaney S, Roberts IJ, Forte M, Hirsh J (2000). Ectopic G-protein expression in dopamine and serotonin neurons blocks cocaine sensitization in *Drosophila melanogaster*. Curr. Biol. CB.

[45] Friggi-Grelin F (2003). Targeted gene expression in *Drosophila* dopaminergic cells using regulatory sequences from tyrosine hydroxylase. J Neurobiol.

[46] Costa M, Calleja M, Alonso CR, Simpson P (2014). The bristle patterning genes hairy and extramacrochaetae regulate the development of structures required for flight in Diptera. Dev. Biol..

